# Distinct contributions of the fornix and inferior longitudinal fasciculus to episodic and semantic autobiographical memory

**DOI:** 10.1016/j.cortex.2017.05.010

**Published:** 2017-09

**Authors:** Carl J. Hodgetts, Mark Postans, Naomi Warne, Alice Varnava, Andrew D. Lawrence, Kim S. Graham

**Affiliations:** a Cardiff University Brain Research Imaging Centre, School of Psychology, Cardiff University, Cardiff, Wales, UK; b BRAIN Unit, Cardiff University, Cardiff, Wales, UK; c MRC Centre for Neuropsychiatric Genetics and Genomics, Division of Psychological Medicine and Clinical Neurosciences, School of Medicine, Cardiff University, Cardiff, Wales, UK

**Keywords:** Hippocampus, Individual differences, Mental time travel, Structural connectivity, Temporal lobe, White matter tractography

## Abstract

Autobiographical memory (AM) is multifaceted, incorporating the vivid retrieval of contextual detail (episodic AM), together with semantic knowledge that infuses meaning and coherence into past events (semantic AM). While neuropsychological evidence highlights a role for the hippocampus and anterior temporal lobe (ATL) in episodic and semantic AM, respectively, it is unclear whether these constitute dissociable large-scale AM networks. We used high angular resolution diffusion-weighted imaging and constrained spherical deconvolution-based tractography to assess white matter microstructure in 27 healthy young adult participants who were asked to recall past experiences using word cues. Inter-individual variation in the microstructure of the fornix (the main hippocampal input/output pathway) related to the amount of episodic, but not semantic, detail in AMs – independent of memory age. Conversely, microstructure of the inferior longitudinal fasciculus, linking occipitotemporal regions with ATL, correlated with semantic, but not episodic, AMs. Further, these significant correlations remained when controlling for hippocampal and ATL grey matter volume, respectively. This striking correlational double dissociation supports the view that distinct, large-scale distributed brain circuits underpin context and concepts in AM.

## 1 Introduction

Reliving our personal history, or autobiographical memory (AM), provides an important form of self-knowledge that is necessary for directing present and future behaviour, forging social bonds, and promoting continuity of the self over time (Bluck, Alea, Habermas, & Rubin, 2005). AM is not a single psychological construct but rather a multifaceted cognitive process involving both episodic and semantic details (Moscovitch et al., 2005, Tulving, 2002). Episodic AM involves remembering past events in a specific spatial and temporal context, and is typically characterised by the vivid retrieval of perceptual and emotional details. Alternatively, semantic AM contains general and self-related knowledge that is independent of the specific spatiotemporal encoding context and is considered to occur in the absence of ‘mental time travel’ (Levine, 2004, Tulving, 2002). Though presumed distinct (Moscovitch et al., 2005), episodic and semantic AM systems are highly interactive and can influence each other (Greenberg and Verfaellie, 2010, Irish and Piguet, 2013). Thus, it has been a major challenge to identify whether these AM components arise from dissociable neural systems.

Functional neuroimaging studies suggest that episodic AM retrieval recruits medial temporal lobe (MTL) but also midline structures, including posteromedial and medial prefrontal cortices (Bonnici et al., 2012, Levine et al., 2004, Martinelli et al., 2013). For example, activity in the hippocampus (HC) has been shown to increase parametrically with the episodic ‘quality’ of AMs (e.g., contextual specificity, emotional vividness), underlining a potential key role of this region in re-experiencing past events (Addis et al., 2004b, Daselaar et al., 2008, Moscovitch et al., 2005). Semantic AM, on the other hand, has been associated with increased activation along the ventrolateral temporal cortex, including anterior temporal lobe (ATL) and occipitotemporal fusiform gyrus (Addis et al., 2004a, Levine, 2004, Martinelli et al., 2013).

To date, neuropsychological studies provide the most compelling evidence for a potential dissociation between episodic/semantic AM. For instance, amnesic patients with MTL damage recall fewer personal episodic details alongside relatively preserved memory for semantic information (Klein and Gangi, 2010, Steinvorth et al., 2005). Further, episodic AM impairment has been shown to be related to the degree of MTL damage in both amnesia (Rosenbaum et al., 2008) and Alzheimer's disease (AD) (Gilboa et al., 2005, Irish et al., 2014). Gilboa et al. (2005) also found that semantic AM was strongly associated with grey and white matter atrophy in ATL and occipital lobe (Gilboa et al., 2005). In contrast, studies of semantic dementia (SD) – a disorder characterised primarily by bilateral degeneration of the ATL (Lambon Ralph, Jefferies, Patterson, & Rogers, 2017) – have found impaired memory for semantic details in AM but preserved memory for specific contextual details (Ivanoiu et al., 2006, Piolino et al., 2003, Westmacott et al., 2001). SD patients can also exhibit better memory for recent events but impaired retrieval of remote AMs (Graham and Hodges, 1997, Irish et al., 2011).

Overall, these studies suggest that the MTL, and in particular the HC, may be critical for the retrieval of episodic, but not semantic information, during real world memory retrieval (but see Klooster and Duff, 2015, Verfaellie et al., 2014). Alternatively, retrieval of semantic details in AM seems dependent on structures along ventrolateral temporal cortex, in particular those regions affected in SD, including ATL.

Despite the evidence cited above, the notion that episodic/semantic AM are underpinned by distinguishable, dissociable neural systems remains controversial (Irish & Piguet, 2013). To date, there has been no demonstration, within the same study, that focal damage to the HC and ATL selectively impacts episodic and semantic AM, respectively. Progressive atrophy in lesion models, such as the effects of HC atrophy in later stages of SD (Maguire et al., 2010, Matuszewski et al., 2009), and of progressive ATL atrophy in AD (Domoto-Reilly, Sapolsky, Brickhouse, & Dickerson, 2012), makes inferences about specific structures, and their association with different AM components, challenging. Critically, despite recognition that AM arises from large-scale network-level communication between brain areas (Andrews-Hanna et al., 2014, Levine, 2004), few studies have directly explored how structural connectivity within broader, distributed brain circuits underpins differences in episodic and semantic AM. While lesion studies suggest the involvement of specific brain structures, it is unclear to what extent this reflects the intrinsic processing of those regions, or wider network-level disruption (Collins et al., 2017, Shamy et al., 2010), particularly given that both episodic and semantic AM appear to engage larger brain networks in healthy controls (Levine et al., 2004, Martinelli et al., 2013).

A novel, network-level approach to testing this potential dissociation is to use diffusion magnetic resonance imaging (dMRI) to examine how inter-individual variation in the microstructure of white matter fibre bundles to and from these putative AM regions predict individual variation in episodic and semantic AM, presumably by influencing the transfer of distinct types of AM content within distributed neural networks (Fields, 2015, Mesulam, 1990). We tested, therefore, whether the tissue microstructural properties of the fornix would relate to the amount of episodic, but not semantic, detail within AMs. The fornix is the major input/output pathway of the HC (see above), and contains axonal projections to the medial prefrontal cortex, mammillary bodies and the anterior thalamic nuclei (Amaral & Lavenex, 2006). Similar to HC lesions, fornix damage in humans causes deficits in episodic recollection (Calabrese et al., 1995, Vann et al., 2009), and diffusion MRI studies show that fornix microstructure predicts episodic memory performance (Metzler-Baddeley, Jones, Belaroussi, Aggleton, & O’Sullivan, 2011). Despite these findings, it is unknown whether fornical microstructure is associated with the ability to recall episodic information within AM. Such a role is feasible given the contribution of other interconnected regions of the so-called “extended HC network” (Gaffan, 1994) to episodic AM, including medial prefrontal cortex (Bonnici et al., 2012).

A second question is whether the amount of semantic information within AMs is less dependent upon an extended HC network, and instead relates to white matter projections to the ATL. The inferior longitudinal fasciculus (ILF) is a large fibre bundle linking occipital lobe with ATL (Bajada et al., 2016). As the major input pathway to this region, the ILF provides a potential anatomical route by which unimodal information from occipitotemporal fusiform gyrus becomes integrated into amodal semantic representations in an ATL-based semantic ‘hub’ (Lambon Ralph et al., 2017). In addition to demonstrations of altered ILF microstructure in SD (Agosta et al., 2010), further clinical and non-clinical studies have reported associations between ILF macro- and micro-structural properties and semantic processing, including verbal comprehension (Ivanova et al., 2016, Wong et al., 2011), word-picture matching (Harvey & Schnur, 2015) and naming (Mehta et al., 2016). It is currently unclear, however, whether inter-individual variation in the ILF supports the provision of semantic information during AM.

To test the contribution of these white matter pathways to episodic and semantic AM, we acquired high angular resolution dMRI data in young healthy participants who were asked to recall AMs using word cues (modified Galton–Crovitz paradigm; Crovitz & Schiffman, 1974). Using an established narrative coding methodology (Levine, Svoboda, Hay, Winocur, & Moscovitch, 2002), we derived subject-specific scores reflecting the total number of episodic and semantic AM details recalled and correlated these with free water corrected measures (see Methods) of white matter tissue microstructure for the fornix and the ILF (fractional anisotropy, FA; mean diffusivity, MD) using constrained spherical deconvolution-based deterministic tractography (Jbabdi & Behrens, 2013). Our main hypothesis was that the microstructural properties of the fornix and ILF are associated with AM retrieval of episodic and semantic information, respectively. Given that increased MD reflects greater diffusion along both axial and radial diffusion directions, and may be associated with reduced conduction velocity along axonal fibres (Beaulieu, 2002), we predicted negative associations between MD and performance (see Hodgetts et al., 2015). Correspondingly, for FA, we predicted a positive association with performance, as this metric reflects the extent to which diffusion within biological tissue is constrained along a single axis, and high FA values may reasonably reflect properties, such as increased myelination, that support efficient information transmission between neural regions (Fields, 2015).

## 2 Materials and methods

### 2.1 Participants

Twenty-seven healthy undergraduates at Cardiff University (2 male; aged 18–22 years; mean = 19; SD = 1) were scanned at the Cardiff University Brain Research Imaging Centre (CUBRIC). Approximately 10 months after the original imaging data acquisition, these participants completed a modified Galton–Crovitz cue word paradigm. Testing was undertaken with the understanding and written consent of each participant. The research was completed in accordance with, and approved by, the Cardiff University School of Psychology Research Ethics Committee.

### 2.2 Autobiographical interview

#### 2.2.1 Procedure

AM was assessed using a modified version of the Galton–Crovitz cue word paradigm (Crovitz & Schiffman, 1974). In this task, participants were provided with 10 cue-words (e.g., “Chair”, “Holiday”) and instructed to produce a detailed and specific autobiographical memory for each word. Three separate word lists were used; these were matched for semantic category (i.e., participants either heard the cue-word ‘holiday’, ‘journey’ or ‘vacation’). Before each testing session, the experimenter would say:

*“In this test I am going to give you a series of words and ask you to produce a memory relating to that word. The memory needs to be as specific and detailed as possible. I would like you to give me as much information as you can. Can you tell me a memory that you have to do with the word ‘Party’?”*

Participants were given approximately 1 min to describe an episode. If the memory was not very specific, or lacked detail, the experimenter would prompt the participant to provide further detail in a non-specific manner (e.g., “Is there anything else you can tell me about this event?”). Participants were not restricted to when, in their lifetime, they could recall memories. A portable recording device (Zoom H1 Digital Field Recorder) was used to record each testing session for subsequent transcription and coding.

#### 2.2.2 Scoring

The recorded memories were transcribed and then segmented and scored using a commonly used, modified version of the Autobiographical Interview coding system (Levine et al., 2002). In order to conduct our planned analyses around the provision of episodic and semantic information, AMs were initially segmented into parts, or ‘details’. Details were typically grammatical clauses referencing a unique occurrence, observation or thought (Levine et al., 2002). As in previous studies, these details were categorised into two broad groups: internal details (details pertaining to the main event) and external details (de-contextualised information, or details not pertaining to the main event). If a participant described more than one event, the event that occurred within the briefest period of time (or that involved the most details) was coded as ‘internal’ and others were coded as ‘external’ (see below for further detail). As the main event was required to be specific in time and place to be coded as internal (i.e., episodic), these details are henceforth described as ‘episodic’ details.

Following initial segmentation, episodic details were subdivided into several subcomponents: event, time, place, perceptual and emotion/thought (see Table 1). Similar to previous studies, the scores for time and place were aggregated to form a spatiotemporal category (Irish et al., 2011). External details were subdivided into semantic, categorical, extended, repetitions, tangential, or other (Table 1). The main external subcomponent, and the focus of our analysis, was semantic, which was defined as factual information or knowledge that was detached from any spatiotemporal context (e.g., “ … cats always go out on their own”). A description of all subcomponents, and example clauses, are contained in Table 1. Scoring was conducted by two raters and a mean of the two scores calculated for each target item. Intra-class correlation (ICC) analysis indicated near perfect agreement between the two raters (see Supplementary Results). Based on the hypotheses outlined in the Introduction, our main variables of interest are the episodic and semantic categories and their relationship with fornix/ILF microstructure.

**Table 1:** **Description of the coding categories used in scoring the modified Galton–Crovitz cue word paradigm**. Examples are provided for each category (episodic/external), and subcomponent (Event, Time, Place, etc.).

Category		Description	Example
**Episodic**	*Event*	Activities, occurrences, actions, people present, reactions in others	Me and my mum went My Nan made me eat some fish He started to have this seizure
	*Time*	Times, dates, days, seasons, years, indications of temporal order of events, frequencies, durations	That was at 8:30 On the Sunday For a few more seconds
	*Place*	Details pertaining to location including country, city/town, area, building, room, area within room, relative positioning to other people/objects	In the Gower To the Aquarium The outside tables
	*Perceptual*	Information perceived from sensory processes. Derived from but not limited to information regarding surroundings, individuals present, other's emotions, distances, weather, temperature.	Which were about an inch thick It was quite windy They were a bit sticky
	*Emotion/Thought*	Feelings and cognitive processes that occurred within the episode	I wasn't planning on doing That really creeped me out I was quite impressed with my mum
**External**	*Semantic*	General and self-related knowledge, facts, opinions	Obviously cats go out on their own He's not got a house at the moment It's one of my favourite cities in the world
	*Categorical*	Any details (event, time, place, perceptual, emotion/thought) regarding repeated episodes of the same activity	When my mother-in-law goes away We get baguettes there whenever we go It's just we sit there and it's always a really awkward conversation
	*Extended*	Any details (event, time, place, perceptual, emotion/thought) regarding an episode that lasts for longer than 1 day or 24 h	We were there for about a week I think, we stayed in a hotel I'm not having a good run of luck with phones at the moment
	*Repetitions*	Details that have been mentioned previously within the episode	N/A
	*Tangential*	Details not related to the main episode or have a weak connection	N/A
	*Other*	Details not covered by other categories, including (but not limited to) retrospective comments about the episode or metacognitive statements	Thinking back on it's quite embarrassing It's probably not my most positive memory This is bad that I've forgotten already

To explore how structure–behaviour correlations were affected by inter-individual differences in the age of memories recalled, AMs were also retrospectively coded as being recent (within the last 6 months) or not (over 6 months; see Sheldon, Farb, Palombo, & Levine, 2016), resulting in a “recency” score reflecting the sum number of recent memories across all 10 narratives (mean = 2.98, median = 2, SD = 2.31, range = 0–9). Coding was determined by time-defining statements in each narrative (e.g., “Last week …”, “At my 15th Birthday …”) relative to the date of testing, which could be accurately extracted in 76% of all subject narratives. An intra-class correlation analysis for the recency measure indicated near perfect agreement between two raters (*r* = .98, *p* < .0001), and the rater-averaged recency scores were used in the correlational analyses.

### 2.3 MRI data acquisition

Imaging data were collected at the Cardiff University Brain Research Imaging Centre (CUBRIC) using a GE 3-T HDx MRI system with an 8-channel receive-only head coil. Whole brain high angular resolution diffusion image (HARDI) data were acquired using a diffusion weighted single-shot spin-echo echo-planar imaging pulse (EPI) sequence with the following parameters: TE = 87 msec; voxel dimensions = 2.4 × 2.4 × 2.4 mm^3^; field of view = 23 × 23 cm^2^; 96 × 96 acquisition matrix; 60 contiguous slices acquired along an oblique–axial plane with 2.4 mm thickness (no gap). To reduce artifacts arising from pulsatile motion, acquisitions were cardiac gated using a peripheral pulse oximeter. Gradients were applied along 30 isotropic directions with *b* = 1200 sec/mm^2^. Three non-diffusion weighted images were acquired with *b* = 0 sec/mm^2^. High-resolution anatomical images were also acquired using a standard T1-weighted 3D FSPGR sequence comprising 178 axial slices (TR/TE = 7.8/3.0 sec, FOV = 256 × 256 × 176 mm, 256 × 256 × 176 data matrix, 20° flip angle, and 1 mm isotropic resolution).

### 2.4 MRI preprocessing

#### 2.4.1 Diffusion MRI

Diffusion MRI data were corrected for distortions resulting from subject head motion and eddy currents using ExploreDTI (Leemans & Jones, 2009). The two-compartment 'Free Water Elimination' (FWE) procedure was then applied *post hoc* to correct for voxel-wise partial volume artifacts arising from free water contamination (Pasternak, Sochen, Gur, Intrator, & Assaf, 2009). Free water contamination (from cerebrospinal fluid) is a particular issue for white matter pathways located near the ventricles (e.g., the fornix), and has been shown to significantly affect tract delineation (Concha, Gross, & Beaulieu, 2005). Following FWE, corrected diffusion indices for FA and MD were computed. FA reflects the extent to which diffusion within biological tissue is anisotropic, or constrained along a single axis, and can range from 0 (fully isotropic) to 1 (fully anisotropic). MD (10^−3^ mm^2^ s^−1^) reflects a combined average of axial diffusion (diffusion along the principal axis) and radial diffusion (diffusion along the orthogonal direction). The resulting free water corrected tissue FA/MD maps were inputs for the tractography analysis.

### 2.5 Tractography

Deterministic whole brain white matter tractography was performed using the ExploreDTI graphical toolbox. Tractography was based on constrained spherical deconvolution (CSD) (Jeurissen, Leemans, Jones, Tournier, & Sijbers, 2011), which extracts peaks in the fibre orientation density function (fODF) at each voxel. By using CSD, multiple peaks can be extracted within each voxel, allowing the representation of crossing/kissing fibres in individual voxels. Each streamline was reconstructed using an fODF amplitude threshold of .1 and a step size of 1 mm, and followed the peak in the fODF that resulted in the smallest step-wise change in orientation. An angle threshold of 30° was used and any streamlines exceeding this threshold were terminated. In particular, tractography methods are well-suited for our specific hypotheses, as they allow tracts of interest to be accurately delineated on the native space diffusion-weighted images of individual participants (i.e., images have not been transformed or co-registered, thus maximising anatomical specificity).

**Fig. 1:**
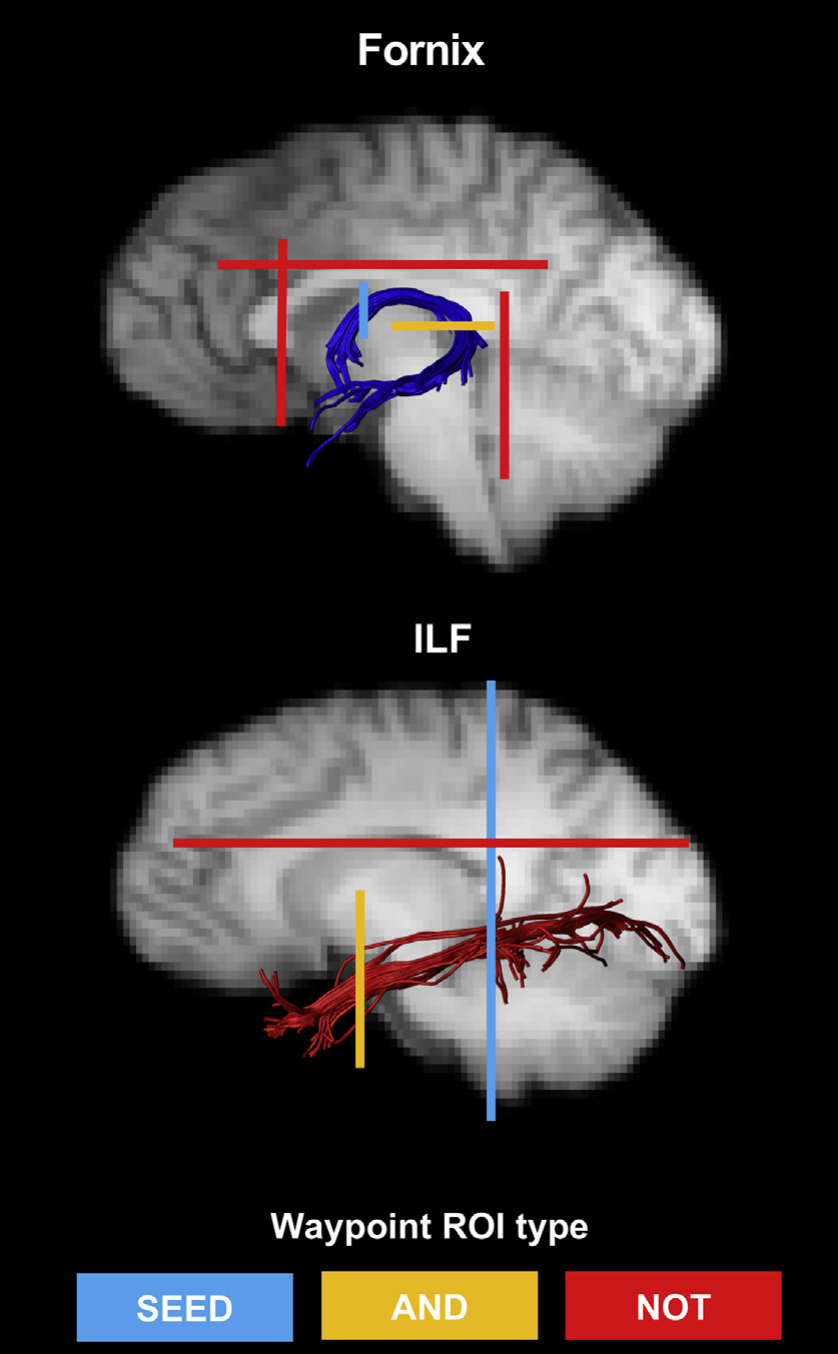
**Example reconstructions for the fornix and inferior longitudinal fasciculus (ILF)**. The waypoint regions-of-interest (ROIs) used for reconstructing each tract are depicted on each image (SEED ROI = Blue; AND ROI = Orange; NOT ROI = Red). The tracts are displayed on sagittal midline slices of a participant's T1-weighted brain image (registered to native diffusion space).

To generate three-dimensional reconstructions of each tract, ‘way-point’ regions-of-interest (ROIs) were manually drawn onto whole-brain FA maps in the diffusion (native) space of individual subjects. In accordance with Boolean logic, these way-point ROIs can specify that: (a) tracts passing through multiple ROIs are retained for analysis (i.e., ‘AND’ ROIs), and (b) tracts passing through certain ROIs are omitted from analysis (i.e., ‘NOT’ ROIs). Depending on the specific tract, or the anatomical plausibility of initial reconstructions, such ROIs can be combined; for example, a tract may pass through ROI-1 ‘AND’ ROI-2 but ‘NOT’ ROI-3. The ROI approaches described below will adopt this Boolean terminology when describing the ROIs that were drawn for each tract. Following the reconstruction of each pathway in each subject, mean MD and FA were calculated by averaging the individual values at each 1 mm step along the tracts. ROIs for each tract are depicted in Fig. 1.

#### 2.5.1 Fornix

A multiple region-of-interest (ROI) approach was adopted to reconstruct the fornix (Metzler-Baddeley et al., 2011). This approach involved placing a seed point ROI on the coronal plane at the point where the anterior pillars enter the fornix body. Using a mid-sagittal plane as a guide, a single AND ROI was positioned on the axial plane, encompassing both crus fornici at the lower part of the splenium of the corpus callosum. Three NOT ROIs were then placed: (1) anterior to the fornix pillars; (2) posterior to the crus fornici; and (3) on the axial plane, intersecting the corpus callosum. Once these ROIs were placed, and the tracts reconstructed, anatomically implausible fibres were removed using additional NOT ROIs.

#### 2.5.2 Inferior longitudinal fasciculus (ILF)

Fibre-tracking of the ILF was performed using a two-ROI approach in each hemisphere (Wakana et al., 2007). First, the posterior edge of the cingulum bundle was identified on the sagittal plane. Reverting to a coronal plane at this position, a SEED ROI was placed that encompassed the whole hemisphere. To isolate streamlines extending towards ATL, a second ROI was drawn at the most posterior coronal slice in which the temporal lobe was not connected to the frontal lobe. Here, an additional AND ROI was drawn around the entire temporal lobe. Similar to the fornix protocol above, any anatomically implausible streamlines were removed using additional NOT ROIs (Fig. 1). This approach was carried out in both hemispheres; diffusion properties of the left and right ILF (for both FA and MD) were averaged across hemispheres to provide a bilateral measure of ILF FA and MD in each participant.

### 2.6 Grey matter volumetrics

Prior to image segmentation/parcellation, T1-weighted images were bias field corrected using FAST (Zhang, Brady, & Smith, 2001). Following this, bilateral grey matter (GM) volume for the HC was derived using FMRIB's Integrated Registration & Segmentation Tool (FIRST; Patenaude, Smith, Kennedy, & Jenkinson, 2012). Bilateral volumes for the ATL were determined using the ventral ATL/temporal pole parcellations from FreeSurfer (Destrieux, Fischl, Dale, & Halgren, 2010; surfer.nmr.mgh.harvard.edu). The resulting individual-level FreeSurfer outputs were quality controlled according to publically available protocols from ENIGMA (http://enigma.ini.usc.edu/). One subject was removed from all correlational analyses due to poor overall data quality on the T1 FSPGR, and a second subject was excluded from the temporal polar correlations because of poor GM segmentation in anterior temporal lobe (*n* = 26 for HC; *n* = 25 for ATL). As temporal lobe substructures have been shown to correlate with intracranial volume (Moran, Lemieux, Kitchen, Fish, & Shorvon, 2001), individual-level ATL and HC volumes were divided by FreeSurfer's estimate of total intracranial volume (eTIV) to create proportional scores (Westman, Aguilar, Muehlboeck, & Simmons, 2013).

## 3 Results

### 3.1 Correlations between tract microstructure and AM

Directional correlations (see Introduction) were conducted between the free water corrected MD and FA values of the fornix and ILF (obtained separately for each participant) and the total number of episodic (mean = 123, median = 113.5, SD = 41.65, range = 60.5–246) and semantic (mean = 11.5, median = 9.5, SD = 7.52, range 1–27.5) details recalled across all narratives (see Supplementary Results). Pearson's correlations were Bonferroni-corrected by dividing *α* = .05 by the number of statistical comparisons for each DTI metric (i.e., .05/2 = .025). We also conducted Bayesian correlation and regression analyses using JASP (https://jasp-stats.org) and the BayesFactor (Morey & Rouder, 2014) and BayesMed (Nuijten, Wetzels, Matzke, Dolan, & Wagenmakers, 2015) packages in R. From this, we report Bayes factors and 95% Bayesian credibility intervals (CI).

**Fig. 2:**
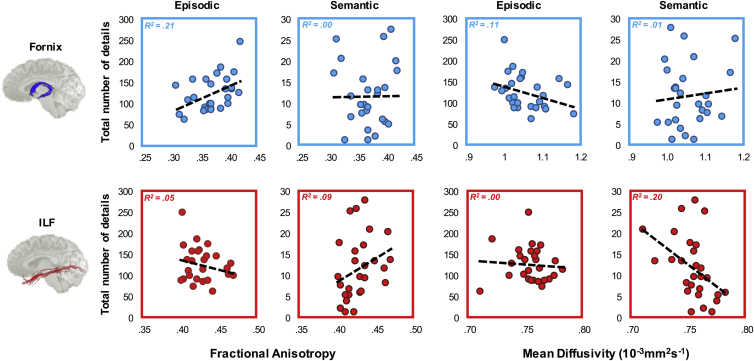
**The relationship between tract diffusion properties and episodic and semantic autobiographical memory**. These results are shown for the fornix (top) and inferior longitudinal fasciculus (ILF, bottom) for each measure (fractional anisotropy, FA, left; mean diffusivity, MD, right). The total number of details recalled (summed across 10 memories), for each AM component, is plotted on the *y*-axis of each plot. The best fitting linear regression line is displayed on each scatter plot. There are 27 data points appearing on each graphs.

The relationship between the diffusion metrics (FA, MD) and the number of episodic/semantic details is presented in Fig. 2. As predicted, we found a significant positive correlation between the number of episodic details retrieved within AMs and fornix FA (*r* = .46, *p* = .01, 95% CI [.1, .7], B_+0_ = 7.46; Fig. 2). There was no relationship between fornix FA and semantic details (*r* = .01; *p* = .48, 95% CI [.01, .41], B_0+_ = 3.97). To determine whether these correlations were significantly different, we conducted a directional Steiger *Z*-test for comparing dependent correlations (Steiger, 1980). The correlation between fornix FA and episodic details was significantly greater than between fornix FA and semantic details (z (24) = 1.85, *p* = .03). We also observed a strong negative trend between fornix MD and the number of episodic details (*r* = −.33, *p* = .05, 95% CI [−.61, .04], B_−0_ = 1.77; Fig. 2). There was no trend between fornix MD and the number of semantic details (*r* = .1; *p* = .31, 95% CI [−.36, −.004], B_0−_ = 5.9; Fig. 2). As shown for fornix FA, above, the association between fornix MD and episodic AM was significantly stronger than the correlation between fornix MD and semantic details (z (24) = 1.74, *p* = .04).

For ILF MD, we found a significant negative correlation with the number of semantic details recalled (*r* = −.44, *p* = .01, 95% CI [−.69, −.09], B_−0_ = 6.1; Fig. 2). In contrast to the fornix data reported above, there was no significant relationship between ILF MD and number of episodic details (*r* = −.07; *p* = .36, 95% CI [−.47, −.01], B_0−_ = 3.02; Fig. 2). There was a trend level difference between these associations (z (24) = 1.51, *p* = .06). While not quite reaching statistical significance, a moderate positive trend was found between ILF FA and semantic details (*r* = .31, *p* = .06, 95% CI [.03, .6], B_+0_ = 1.43; Fig. 2). A weak, but non-significant, negative association was evident between ILF FA and number of episodic details recalled (*r* = −.23, *p* = .13, 95% CI [.003, .29], B_0+_ = 8.33; Fig. 2). The Steiger *Z*-test analysis confirmed a significant difference between these two correlations (z (24) = 2.17, *p* = .02). Nonparametric correlations are reported in the Supplementary Results.

**Fig. 3:**
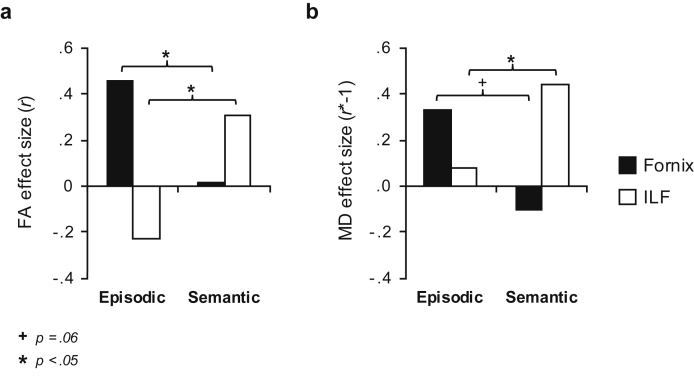
**Effect size comparison for episodic and semantic autobiographical memory and fornix/inferior longitudinal fasciculus (ILF) microstructure**. These data are plotted for (a) fractional anisotropy (FA) and (b) mean diffusivity (MD). Effects (Pearson's *r* values) for the fornix and ILF are indicated by black and white bars, respectively. To aid comparison across the two microstructural metrics, the MD coefficients are reversed. The asterisks depict significant differences between correlation coefficients as determined by the Steiger *Z*-test (see Results).

For visualisation purposes, we plot the coefficients for the fornix and ILF correlations in Fig. 3. This plot highlights that while effect sizes were found to vary across diffusion measures, both FA and MD provide converging evidence regarding the relative contribution of the fornix and ILF to episodic and semantic AM, respectively. Exploratory voxel-wise analyses testing any potential associations with episodic/semantic AM outside our main tracts-of-interest (TOIs) are reported in the Supplementary Results and Supplementary Figure S1.

### 3.2 Inter-hemispheric differences in ILF-semantic correlations

Given neuropsychological evidence that general semantic memory is left lateralised, we also tested whether the association between semantic AM would be stronger in left compared to right ILF. There was indication for a stronger relationship between ILF microstructure and semantic AM details in the left hemisphere, which is most pronounced for FA (*left ILF FA*: *r* = .4, *p* = .02, 95% CI [.06, .65], B_+0_ = 3.34; *right ILF FA*: *r* = .09, *p* = .66, 95% CI [.01, .46], B_0+_ = 2.9). There was likewise a numerically stronger relationship in the left versus right for ILF MD (*left ILF MD*: *r* = −.48, *p* = .01, 95% CI [−.71, −.12], B_−0_ = 10.04; *right ILF MD*: *r* = −.31, *p* = .06, 95% CI [−.6, −.03], B_−0_ = 1.44). Despite this trend, there were no significant inter-hemispheric differences for either FA (z (24) = 1.35, *p* = .09) or MD (z (24) = .57, *p* = .28) in their association with semantic AM, consistent with the view that semantic knowledge is represented bilaterally in the ATLs but may show subtle inter-hemispheric (left > right) gradations for verbal stimuli (Rice, Lambon Ralph, & Hoffman, 2015).

### 3.3 Controlling for memory age

One potential issue with these results is the possibility that AMs with greater episodic detail are also more recent (Grilli & Verfaellie, 2016). As such, the reported significant association between fornix tissue microstructure and episodic AM could reflect individual differences in the age of memories recalled, and not episodic detail *per se*. As subjects' memories were not constrained to specific time periods, AMs were retrospectively coded as recent (within the last 6 months) or remote (over 6 months; see Sheldon et al., 2016), resulting in a recency score per subject (see Methods). An intra-class correlation analysis for the recency measure indicated near perfect agreement between two raters (*r* = .98, *p* < .0001). Partial correlations were conducted to show that fornix white matter microstructure is associated with episodic AM over and above its potential contribution to recent AMs. When controlling for recency, a significant correlation between episodic AM and fornix FA was observed (*r* = −.46, *p* = .01, 95% CI [.1, .78]; B_+0_ = 7.42), consistent with the initial analysis. There was no significant association between fornix FA and semantic AM when controlling for memory recency (*r* = .02, *p* = .47, 95% CI [.01, .46]; B_0+_ = 4.22). Likewise, we observed a moderate trend between fornix MD and episodic AM (*r* = −.35, *p* = .04, 95% CI [−.7, −.04]; B_−0_ = 1.74) and a weak positive association between fornix MD and semantic AM (*r* = .1, *p* = .31, 95% CI [−.4, .004]; B_0−_ = 6.29).

When controlling for memory recency in the ILF correlations, we likewise found a significant negative correlation between ILF MD and semantic AM (*r* = −.45, *p* = .01, 95% CI [−.77, −.09]; B_−0_ = 6.26) and no significant association between ILF MD and episodic AM (*r* = −.08, *p* = .35, 95% CI [−.5, .01]; B_0−_ = 3.22). We found a moderate association between ILF FA and semantic AM when controlling for recency (*r* = .32, *p* = .05, 95% CI [.04, .71]; B_+0_ = 1.5). A weak negative trend was found between ILF FA and episodic AM (*r* = −.24, *p* = .12, 95% CI [.34, .003]; B_0+_ = 9.43).

These results demonstrate that subjects' preference for recalling recent versus remote AMs does not account for the distinct contributions of the fornix and ILF microstructure to episodic and semantic AM, respectively.

### 3.4 Influence of HC and ATL volume

A second key question was whether these correlations remain when controlling for regional grey matter volume. To address this, we conducted partial correlations to see whether the significant *fornix-episodic AM* and *ILF-semantic AM* correlations remain when controlling for hippocampal and temporal polar volume, respectively (see Methods).

When hippocampal volume (corrected for estimated total intracranial volume – eTIV) was controlled for, the significant association (one-tailed) between episodic AM and fornix FA was still observed, and became slightly stronger (*r* = .47, *p* < .01, 95% CI [.1, .8]; B_+0_ = 7.81). Likewise, a moderate trend between fornix MD and episodic AM was still found when controlling for hippocampal volume (*r* = −.36, *p* = .04, 95% CI [−.72, −.04]; B_−0_ = 2). For the ILF, the significant negative relationship between MD and semantic AM remained when controlling for ventral ATL/temporal pole volume (*r* = −.46, *p* = .01, 95% CI [−.79, −.09], B_−0_ = 5.9). For ILF FA, a moderate positive trend with semantic AM was seen (*r* = .25, *p* = .12, 95% CI [.02, .6], B_+0_ = .8).

### 3.5 Fornix microstructure and episodic AM subcomponents

Based on the key hypotheses outlined in the Introduction, we have focused on the association between fornix microstructure and episodic AM more broadly (i.e., the summed total of each subcomponent). To explore whether a particular episodic subcomponent (event, spatiotemporal, etc.) better predicts inter-individual variation of fornix microstructure, we conducted Bayesian linear regression analysis (https://jasp-stats.org). Here, free water corrected FA and MD values for the fornix were entered as dependent variables, and the episodic subcomponents (event, spatiotemporal, perceptual, emotion/thought) were entered as predictors. This analysis revealed that the number of spatiotemporal details was the best predictor of fornix FA relative to all other models (spatiotemporal BM = 3.92; all other models < 1.61). Relative to the null (B_10_), we find substantial evidence for the spatiotemporal model (BF_10_ = 4.99; all other models < 2.33). Additional exploratory whole brain analyses relating to these episodic subcomponents can be found in the Supplementary Results.

## 4 Discussion

We tested the hypothesis that microstructure of the major white matter fibre tracts converging on the HC (fornix) and ATL (ILF) would be differentially associated with the number of episodic and semantic details recalled, respectively, during cued autobiographical retrieval in healthy adults. We found that inter-individual variation in the microstructural properties (FA, MD) of the fornix was associated with the number of episodic, but not semantic, details recalled within AMs. In contrast, ILF microstructure was associated with the amount of semantic, but not episodic, detail in AMs. Importantly, the episodic and semantic AM correlations were found to differ statistically for both the fornix and ILF, confirming a double dissociation between these two pathways and their contribution to episodic and semantic AM (Nieuwenhuis, Forstmann, & Wagenmakers, 2011).

The finding that the main input/output pathway of the HC (the fornix) is related to episodic, but not semantic, AMs, is consistent with the functional role of the HC, and its extended network, in the episodic retrieval of personal past events (Moscovitch et al., 2005). In particular, these dMRI findings closely converge with studies showing episodic, but not semantic, AM impairments in patients with focal HC lesions (Klein and Gangi, 2010, Steinvorth et al., 2005), but also in AD (Gilboa et al., 2005, Murphy et al., 2008). Similarly, functional neuroimaging studies have shown that episodic AM, when contrasted directly with semantic AM conditions, results in increased HC activity (Gilboa et al., 2004, Levine et al., 2004, Martinelli et al., 2013). Further, parametric increases in specific ‘episodic’ features (e.g., emotionality, contextual specificity) appears to increase HC involvement, suggesting a role of the HC in representing episodic details in personal memories (Addis et al., 2004b, Daselaar et al., 2008).

Here, we go beyond such investigations by demonstrating that the retrieval of episodic details in AM is not a property of the HC *per se*, but may be driven by interactions within a broader, extended hippocampal network, which critically involves its extrinsic connectivity with cortical and sub-cortical brain areas (Gaffan, 1994). This was supported, in particular, by the finding that fornix microstructure correlated with episodic AM even when controlling for inter-individual differences in HC volume (see also Shamy et al., 2010). The broader involvement of an 'extended HC network' is supported by co-activation of brain areas during episodic AM that are strongly connected to the HC via the fornix. The thalamus, for instance, receives both direct (via the fornix) and indirect (via the mammilothalamic tract) efferents from the subiculum of the HC (Amaral & Lavenex, 2006), and has been shown to respond during specific versus general AM (Holland, Addis, & Kensinger, 2011) and during episodic AM (Addis et al., 2004b, Levine et al., 2004). Likewise, both HC and medial prefrontal cortex (mPFC) show a relatively greater response during episodic versus semantic AM tasks (Addis et al., 2004a, Levine et al., 2004), and connectivity between these regions at rest is correlated with episodic AM (Yang, Bossmann, Schiffhauer, Jordan, & Immordino-Yang, 2012). A further study found that multivariate patterns of activation in both mPFC and HC contain information about episodic AMs (Bonnici et al., 2012). Another recent study looking at theta-phase synchrony during AM retrieval found that the MTL was phase synchronised with the mPFC (Fuentemilla, Barnes, Düzel, & Levine, 2014). Moreover, this synchrony was higher during the re-experiencing of episodic versus general semantic information, indicating that HC-mPFC interactions may be important for the vivid retrieval of episodic AMs. These findings are particularly striking given that the strong reciprocal connections that the HC forms with mPFC may be mediated entirely by fornical connections (Amaral & Lavenex, 2006).

While the involvement of the fornix in AM has received only limited study, one notable study described an individual with fornix damage who, when presented with family photographs, was able to provide personal semantic details but could not provide detailed contextual information (Poreh et al., 2006). Another case (Vann et al., 2008) with fornix and septal damage similarly showed impaired recall of specific autobiographical incidents, but relatively intact personal semantic memory. Perhaps more strikingly, direct electrical stimulation (DES) in the region of the fornix/hypothalamus has been shown to induce déjà vu episodes involving the involuntary re-experiencing of specific past events (Hamani et al., 2008). The findings presented here extend these results by demonstrating, in the healthy intact brain, the behavioural relevance of fornix white matter microstructure in determining the episodic richness of everyday memories.

Our finding that fornix tissue microstructure was most strongly related to the retrieval of spatiotemporal details highlights the potential role of this extended HC system in contextual processing. Indeed, fornix transection in animals has been shown to impair spatial scene learning in animals (Gaffan, 1994), and dMRI studies in healthy participants have shown that inter-individual variability in fornix microstructure is related to visual scene, but not face, discrimination (Hodgetts et al., 2015, Postans et al., 2014). The shared contribution of this extended HC system (including the fornix) to episodic memory and spatial scene processing serves to underline the fundamental role of spatiotemporal context in episodic AM, and supports the broader view that HC contributions to episodic memory are potentially explained by the encoding and/or reinstatement of spatiotemporal context (Eichenbaum & Cohen, 2014).

A potential confound in this study was the possibility that AMs with greater episodic detail were also more recent (Grilli & Verfaellie, 2016). Partial correlation analyses demonstrated that the association between fornix microstructure and episodic AM was still significant after controlling for subjects' preference for recalling recent versus remote memories. This is consistent with previous studies that have demonstrated that the recollective quality of memories are better predictors of HC involvement than memory recency *per se* (Addis et al., 2004b, Bonnici et al., 2012, Sheldon and Levine, 2013).

Strikingly, individual variation in ILF microstructure was related to the amount of semantic, but not episodic, detail generated within AMs. The ILF is a long-range cortical association tract running the length of the temporal lobe, connecting the occipital lobe with anterior regions of the middle and inferior temporal gyri, terminating in the ATL (Bajada et al., 2016, Catani et al., 2003). This dissociation mirrors that seen in patients with SD, who show impairments in semantic AM (as part of a generalised impairment of semantic cognition) but relatively preserved episodic AM (Ivanoiu et al., 2006, Piolino et al., 2003, Westmacott et al., 2001; but see; Irish & Piguet, 2013). SD is thought to arise from neurodegeneration of ATL bilaterally (Lambon Ralph et al., 2017). Within a distributed ‘semantic network’, the ATL has been proposed to act as a “hub” that integrates modality-specific information to create transmodal representations (Lambon Ralph et al., 2017). The ILF may be critical for bidirectional interactions between the ATL hub and representations supported by occipital and middle/posterior temporal regions. Indeed, damage to the (left) ILF has been shown to correlate with semantic impairments in SD (Agosta et al., 2010) and following other forms of brain damage, including stroke, head trauma and surgical resection (Herbet et al., 2016, Ivanova et al., 2016, Xing et al., 2017). This is true even when controlling for the extent of cortical damage in ATL (Mehta et al., 2016).

These findings serve to emphasise the critical importance of long-range connectivity along the temporal lobes, mediated by ILF, in semantic AM, and semantic cognition more broadly (see also Chen, Lambon Ralph, & Rogers, 2017). This may account for findings from functional neuroimaging studies that have reported co-activation/functional connectivity of ATL and more posterior ventral temporal and occipital regions during semantic AM (Maguire et al., 2001, Martinelli et al., 2013) and other semantic tasks (Binder, Desai, Graves, & Conant, 2009). These results suggest that the ILF is a key WM fibre tract mediating such coordinated activity. In addition, a critical role for ILF in semantic cognition may explain why large lesions of middle temporal gyrus result in profound semantic impairments, since communication across the length of the temporal lobe would be disrupted as a result of ILF damage (Turken & Dronkers, 2011).

We also conducted exploratory whole brain voxel-wise analyses (see Supplementary Methods & Results) to explore any potential associations outside our key tracts of interest. This identified associations between semantic AM and microstructural variation in frontal and temporo-frontal white matter tracts (inferior fronto-occipital fasciculus, forceps minor, uncinate fasciculus) that have previously been associated with semantic control (Duffau et al., 2013, Lambon Ralph et al., 2017, Moritz-Gasser et al., 2013). A whole brain analysis of the episodic subcomponents also identified significant associations between the amount of emotion/thought detail in AMs and microstructure within mPFC, but also along the ILF and temporal pole – regions previously implicated in emotional concept processing (Rice et al., 2015) and processing self/other mental states (Andrews-Hanna et al., 2014).

Our study has some limitations. Firstly, our sample size was relatively modest. Nevertheless, as indicated by the reported Bayes factors, our study has strong evidential value (Wagenmakers et al., 2015). Secondly, it is not possible to determine whether our results reflect influences of white matter microstructural variation on processes operating during initial encoding and/or retrieval of AMs. Recent proposals highlight bidirectional frontal–hippocampal interactions (potentially mediated by the fornix) that support both the ability to create rich contextual representations underpinning episodic memories, and also use these contextual representations at retrieval (Preston & Eichenbaum, 2013). Similarly, variation in ILF microstructure could impact both ‘bottom-up’ influences on the encoding of semantic representations in the ATL as well as ‘top-down’ semantic influences on lower level sensory cortices (Binney, Parker, & Lambon Ralph, 2012).

Finally, biological interpretation of individual differences in diffusion measures is challenging. While both diffusion measures (FA & MD) provided converging evidence, we did find stronger effects in the tractography analysis for FA in the fornix, and MD in the ILF, thus highlighting the importance of examining more than one diffusion parameter (see also Johnson et al., 2014). Variation in such measures could arise from several underlying functionally relevant biological properties, including axon density, axon diameter, myelination, and the manner in which fibres are arranged in a voxel (Beaulieu, 2002). A recent study, for instance, found strong correspondence between myelin microstructure and DTI microstructural indices, where high FA was linked to high myelin density and the histological orientation, whereas high MD was related to diffuse histological orientation and low myelin density (Seehaus et al., 2015). Critically, such underlying microstructural properties are important for facilitating information transmission between distributed neural regions. For instance, activity-dependent variation in axon myelination may support functional coupling between distal brain regions by regulating conduction velocities (Fields, 2015, Pajevic et al., 2014).

### 4.1 Conclusions

In summary, we found that individual differences in the microstructural properties of the fornix predicted the episodic richness of AMs, but not the amount of semantic detail. This result is consistent with a role for an extended HC network, interconnected via the fornix, in the encoding and reconstruction of spatiotemporal context in AM. Confirming a double dissociation, the microstructure of the ILF – linking fusiform cortex and ATL – was associated with inter-individual variation in the amount of semantic, but not episodic, detail expressed within AMs. This is consistent with recent proposals that the ILF is a critical path in a distributed semantic network anchored by an ATL hub. Broadly, these results highlight how individual differences in the way we re-experience our personal past, based around the ability to access contextual and conceptual content, are shaped by variation within dissociable, large-scale neuroanatomical brain circuits.

## Author contributions

C.J.H., K.S.G. and A.D.L. contributed to the conception and design of the experiment; C.J.H. and M.P. contributed to data acquisition; All authors contributed to data analysis and interpretation. All authors contributed to drafting and revising the manuscript. A.D.L. and K.S.G. jointly supervised this work.

## Funding

This work was supported by funds from the Medical Research Council (MRC; G1002149; KSG, CJH, AV), a Wellcome Trust Strategic Award (104943/Z/14/Z; CJH), a Wellcome Trust Institutional Strategic Support Fund (CJH), Waterloo Foundation (KSG), Biotechnology and Biological Sciences Research Council (BBSRC; BB/I007091/1; KSG, MP) and an Experimental Psychology Society Undergraduate Research Bursary (NW).
