# Supplementary material for: Distinct contributions of the fornix and inferior longitudinal fasciculus to episodic and semantic autobiographical memory
Source: Cortex. 2017 Sep;94:1–14. doi: 10.1016/j.cortex.2017.05.010 (PMC5576916; doi:10.1016/j.cortex.2017.05.010)
Supplement: Supplementary file 1 [file mmc1.docx]

**Supplementary material for “Distinct contributions of the fornix and inferior longitudinal fasciculus to episodic and semantic autobiographical memory”**

Carl J. Hodgetts*, Mark Postans, Naomi Warne, Alice Varnava, & Andrew D. Lawrence^+^, & Kim S. Graham^+1,2^

^1^ Cardiff University Brain Research Imaging Centre, School of Psychology, Cardiff University, Maindy Road, Cardiff CF24 4HQ, Wales, UK

^2^ Neuroscience and Mental Health Research Institute, Hadyn Ellis Building, Cardiff CF24 4HQ, Wales, UK

**This file contains:**

Supplementary Figures

Supplementary Tables

Supplementary Methods

Supplementary Results

Supplementary References

**Supplementary Figures**

**Supplementary Figure S1**

**Supplementary Figure S1. Results of the whole brain tract-based spatial statistics (TBSS) analysis.** (a) Whole brain mean diffusivity (MD) clusters reflecting a significantly greater negative association for semantic > episodic autobiographical memory. (b) Fractional anisotropy (FA) clusters reflecting a significantly greater association for semantic > episodic autobiographical memory. Abbreviated white matter structures include anterior thalamic radiation (ATR) and IFOF (inferior fronto-occipital fasciculus). A table of peak coordinates for this whole brain analysis can be found in Supplementary Table S2. Significant clusters in both panels were visualised and 'thickened' using 'TBSS fill' in FSL.

**Supplementary Figure S2**

**Supplementary Figure S2.** Additional results for the whole brain tract-based

spatial statistics (TBSS) analysis. (a) Whole brain mean diffusivity (MD) clusters

reflecting a significant negative association with emotion/thought details. (b) Whole

brain fractional anisotropy (FA) clusters reflecting a significant positive association

with the emotion/thought component. Abbreviated white matter structures include

IFOF (inferior fronto-occipital fasciculus), inferior longitudinal fasciculus (ILF) and

corpus callosum (CC). Significant clusters in both panels were visualised and

'thickened' using 'TBSS fill' in FSL.

**Supplementary Tables**

**Supplementary Table S1. Table of descriptive statistics for each autobiographical memory subcomponent.** Mean, standard deviation (SD), standard error (SE) and the range are shown for each main category (episodic, external) and their subcomponents (Spat = spatiotemporal; Pcpt = perceptual; Em/T = emotion/thought; Sem = semantic; Cat = categorical; Ext = extended; Rep = repetitions; Tang = tangential).

|  | ***Episodic*** | | | | | ***External*** | | | | | | |
| --- | --- | --- | --- | --- | --- | --- | --- | --- | --- | --- | --- | --- |
|  | *Overall* | *Event* | *Spat* | *Pcpt* | *Em/T* | *Overall* | *Sem* | *Cat* | *Ext* | *Rep* | *Tang* | *Other* |
| **Mean** | 123.0 | 54.6 | 32.4 | 21.1 | 14.9 | 73.4 | 11.5 | 20.2 | 27.9 | 5.0 | 0.8 | 8.0 |
| **SD** | 41.7 | 18.9 | 10.2 | 12.0 | 8.0 | 39.2 | 7.5 | 18.5 | 17.1 | 3.9 | 1.7 | 5.5 |
| **SE** | 8.0 | 3.6 | 2.0 | 2.3 | 1.5 | 7.5 | 1.4 | 3.6 | 3.3 | 0.7 | 0.3 | 1.1 |
| **Min** | 60.5 | 26.5 | 18.0 | 3.0 | 4.0 | 22.0 | 1.0 | 0.0 | 1.5 | 0.5 | 0.0 | 1.0 |
| **Max** | 246.0 | 109.5 | 55.5 | 54.5 | 34.5 | 186.5 | 27.5 | 70.5 | 70.0 | 16.5 | 7.5 | 24.0 |
| **Range** | 185.5 | 83.0 | 37.5 | 51.5 | 30.5 | 164.5 | 26.5 | 70.5 | 68.5 | 16.0 | 7.5 | 23.0 |

**Supplementary Table S2. Whole brain peak co-ordinates for the tract-based spatial statistics (TBSS) analysis of episodic and semantic autobiographical memory.** Clusters are listed for mean diffusivity (MD, top) and fractional anisotropy (FA, bottom) based on a corrected cluster threshold of p = 0.05. The location of these clusters is presented in Supplementary Figure S1.

| ***Whole brain MD*** | |  |  |  |  |  |
| --- | --- | --- | --- | --- | --- | --- |
| ***Semantic > Episodic*** | | |  |  |  |  |
| **Cluster** | **Voxels** | **Z** | **x** | **y** | **z** | **Region** |
| 1 | 5374 | 0.014 | -19 | 48 | 6 | ***forceps minor/IFOF*** |
| 2 | 91 | 0.044 | 18 | -81 | -5 | ***IFOF*** |
|  |  |  |  |  |  |  |
| ***Whole brain FA*** | |  |  |  |  |  |
| ***Semantic > Episodic*** | | |  |  |  |  |
| **Cluster** | **Voxels** | **Z** | **x** | **y** | **z** | **Region** |
| 1 | 490 | 0.042 | -17 | 37 | 7 | ***forceps minor/callosal body*** |
| 2 | 21 | 0.049 | -26 | 19 | 17 | ***IFOF*** |
| 3 | 18 | 0.049 | -26 | 27 | 12 | ***IFOF*** |

**Supplementary Table S3. Whole brain peak co-ordinates for the tract-based spatial statistics (TBSS) analysis of the episodic autobiographical memory subcomponents.** White matter (WM) cluster peaks are reported for mean diffusivity (MD, top) and fractional anisotropy (FA, bottom) with a corrected cluster threshold of p = 0.05. No significant whole brain clusters were found for event, spatiotemporal and perceptual details. Abbreviated white matter structures include ILF (inferior longitudinal fasciculus), IFOF (inferior fronto-occipital fasciculus) and SLF (superior longitudinal fasciculus).

| ***Whole brain MD*** | |  |  |  |  |  |
| --- | --- | --- | --- | --- | --- | --- |
| ***Emotion/Thought*** | |  |  |  |  |  |
| **Cluster** | **Voxels** | **Z** | **x** | **y** | **z** | **Region** |
| 1 | 6318 | 0.01 | -17 | 35 | 11 | *forceps minor* |
| 2 | 4175 | 0.027 | 30 | 43 | -2 | *IFOF* |
| 3 | 2850 | 0.037 | 18 | -76 | 16 | *optic radiation* |
| 4 | 1927 | 0.034 | -21 | -57 | 28 | *callosal body* |
| 5 | 1559 | 0.04 | -44 | -15 | -19 | *ILF (anterior)* |
| 6 | 210 | 0.048 | 42 | -39 | 25 | *SLF* |
| 7 | 203 | 0.048 | -38 | -52 | -13 | *ILF (posterior)* |
| 8 | 179 | 0.049 | -28 | 5 | -35 | *temporal pole WM* |
| 9 | 47 | 0.05 | -41 | -49 | -1 | *ILF (posterior)* |
| 10 | 26 | 0.05 | -25 | -43 | 45 | *primary somatosensory cortex WM* |
| 11 | 20 | 0.05 | -37 | -50 | -6 | *ILF (anterior)* |
| 12 | 3 | 0.05 | -42 | -49 | 6 | *SLF* |
|  |  |  |  |  |  |  |
| ***Whole brain FA*** | |  |  |  |  |  |
| ***Emotion/Thought*** | |  |  |  |  |  |
| **Cluster** | **Voxels** | **Z** | **x** | **y** | **z** | **Region** |
| 1 | 284 | 0.041 | 10 | 27 | -6 | *callosal body* |
| 2 | 213 | 0.045 | 17 | -3 | 36 | *callosal body* |
| 3 | 122 | 0.044 | 29 | -15 | 50 | *corticospinal tract* |
| 4 | 25 | 0.047 | 43 | -22 | 49 | *postcentral gyrus WM* |
| 5 | 9 | 0.05 | 33 | -16 | 57 | *precentral gyrus WM* |
| 6 | 5 | 0.05 | 33 | -29 | 45 | *postcentral gyrus WM* |

**Supplementary Methods**

***Tract-Based Spatial Statistics (TBSS)***

Voxel-wise statistical analysis of the diffusion tensor imaging (DTI) data was carried out using TBSS (Smith et al., 2006). TBSS allows for voxel-wise correlations by non-linearly projecting subjects' DTI metrics (e.g., fractional anisotropy, FA, or mean diffusivity, MD) onto a mean tract skeleton and registering this to a standard template (Montreal Neurological Institute (MNI) 152 1mm template). This approach is valuable in the absence of specific anatomical hypothesis (such as the whole brain analyses described below) but lacks the anatomical specificity of tractography methods (see main text) as voxels in the tract skeleton may correspond to different tracts, or tract regions, in individual brains. Further, while tractography involves averaging diffusion indices along both tracts and streamlines, resulting in a single metric for each measure, voxel-wise methods require correlations at each point of the skeleton, thus reducing statistical power.

To investigate the relationship between inter-individual variation in diffusion properties (FA, MD) across the whole brain and autobiographical memory (AM), we applied a general linear model (GLM) with two explanatory variables: (1) the number of episodic details, and (2) the number of semantic details recalled. To control for individual differences in participants’ preference for recalling more recent memories, we also added memory recency scores as a covariate of no interest (see main text). Direct contrasts (controlling for memory recency) were performed to identify voxels reflecting a stronger association with episodic versus semantic details (episodic > semantic), and *vice versa* (semantic > episodic) for both FA and MD. A second model was constructed to test for positive (FA) and negative (MD) associations between each episodic subcomponent (event, spatiotemporal, perceptual, emotion/thought) and tract microstructure. To address the possibility of reporting false-positives (see Methods), whole brain clusters (for each model) were extracted with a family-wise error (FWE) corrected threshold of p = 0.05. All reported TBSS co-ordinates are in MNI 152 space.

**Supplementary results**

***Inter-rater reliability***

An intra-class correlation analysis (ICC; two-way random model conducted in IBM SPSS statistics 20) was conducted to evaluate the inter-rater consistency across all AM sub-categories and indicated near perfect agreement between the two raters. Significant ICCs (all ps < 0.01) were observed for both the main categories (episodic: r = 0.99; external: r = 1.0) and their respective subcomponents (episodic: event r = 0.99; time r = 0.98; place r = 0.97; perceptual r = 0.99; emotion/thought r = 0.99; external: semantic r = 0.99; categorical r = 0.98; extended r =.97; repetition r =.98; tangential r =.95; other r =.97).

***Autobiographical memories***

Prior to our main analyses (see main text), we will first describe the general characteristics of the data across the two main AM categories (episodic & external). Descriptive statistics for these categories - and their respective subcomponents - are presented in Supplementary Table S1. As can be seen, participants recalled more episodic details compared to external details, though scores for both categories were equally variable across participants (e*pisodic*: mean = 123, SD = 41.7; *external*: mean = 73.4, SD = 40). A within-subjects t-test between these conditions confirmed a significant difference between these categories (t (1, 26) = 5.45, p < 0.01). Within the episodic category, individuals recalled a greater number of event details (i.e., what happened) compared to other episodic details, as observed in other studies (Irish et al., 2011; Levine et al., 2002). A within-subjects ANOVA for episodic details revealed a main effect of subcomponent (F (3, 78) = 104.3, p < 0.01). Bonferroni-corrected pair-wise t-tests revealed significant differences between all subcomponents (event > spatiotemporal > perceptual > emotion/thought; all ps < 0.05).

There were also differences between the external subcomponents, with the majority of external details being classified as 'extended' (i.e., descriptions of events lasting longer than 24 hours; for example responses, see Table 1, main manuscript).

***Non-parametric correlations between tract microstructure and AM***

We also conducted complementary one-tailed Kendall’s
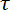
 rank tests for our key correlations (see main text), which are less sensitive to potential outliers. Spearman’s correlations were Bonferroni-corrected by dividing α = 0.05 by the number of statistical comparisons for each DTI metric (i.e., 0.05/2 = 0.025). Bayes factors and 95% Bayesian credibility intervals (CI) are also reported (see Supplementary Methods). A significant association with fornix FA was found for episodic AM (
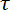
 = 0.46, p = 0.01, 95% CI [0.06, 0.53], B_+0_ = 6.98), but not semantic AM (
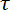
 = 0.01, p = 0.47, 95% CI [0.004, 0.31], B_0+_ = 3.59). A strong trend was found between fornix MD and episodic AM (
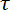
 = -0.33, p = 0.03, 95% CI [-0.49, -0.04], B_-0_ = 2.9) but not semantic AM (
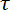
 = 0.1, p = 0.47, 95% CI [-0.28, -0.004], B_0-_ = 4.11). For the ILF, a significant negative relationship was found between MD and semantic AM (
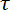
 = -0.44, p = 0.01, 95% CI [-0.65, -0.02], B_-0_ = 3.59). No significant association was found between ILF MD and episodic AM (
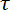
 = -0.08, p = 0.35, 95% CI [-0.38, -0.01], B_0-_ = 14.67). There was a significant positive correlation between ILF FA and semantic AM (
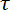
 = 0.31, p = 0.06, 95% CI [0.04, 0.5], B_+0_ = 3.51), but only a weak negative association with episodic AM (
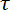
 = -0.22, p = 0.87, 95% CI [-0.48, 0.24], B_0+_ = 7.23)

***Voxel-wise approach***

*Exploratory whole brain analysis of episodic/semantic AM*

We conducted an exploratory whole brain analysis using TBSS to investigate any potential voxel-wise correlations with episodic and semantic AM outside our main tracts-of-interest. The direct contrast reflecting a significant greater negative association for semantic versus episodic and MD revealed a large cluster in forceps minor, extending into the frontal termini of the left inferior fronto-occipital fasciculus (IFOF) and left uncinate fasciculus (-19, 48, 6, p = 0.01, 5374 voxels). A second smaller cluster was found in right posterior IFOF (18, -81, -5, p = 0.04). These are depicted in Supplementary Figure S1. No whole brain clusters were found for the episodic > semantic contrast.

When these contrasts were applied to the FA data, a cluster in left forceps minor/corpus callosum (genual) was found for semantic > episodic AM (-17, 37, 7, p = 0.05, 490 voxels; Supplementary Figure S1). Two smaller clusters were identified in left IFOF (see Supplementary Table S2). There were no whole brain voxels for the episodic AM contrast.

*Whole brain analysis of episodic AM subcomponents*

To explore the relationship between the remaining episodic subcomponents and diffusion properties, we conducted an additional whole brain GLM that included event, spatiotemporal, perceptual and emotion/thought. For MD, we found no clusters reflecting a negative association with event, spatiotemporal or perceptual details. For emotion/thought, however, we identified 12 significant clusters (Supplementary Table S3). The peak cluster was located in left forceps minor (-17, 37, 6, p = 0.01), with sub-peak clusters located in right frontal white matter, corpus callosum, ILF and SLF (Supplementary Figure S2).

**Supplementary References**

Dienes, Z., 2014. Using Bayes to get the most out of non-significant results. Front. Psychol. 5, 1–17. doi:10.3389/fpsyg.2014.00781

Irish, M., Hornberger, M., Lah, S., Miller, L., Pengas, G., Nestor, P.J., Hodges, J.R., Piguet, O., 2011. Profiles of recent autobiographical memory retrieval in semantic dementia, behavioural-variant frontotemporal dementia, and Alzheimer’s disease. Neuropsychologia 49, 2694–702. doi:10.1016/j.neuropsychologia.2011.05.017

Levine, B., Svoboda, E., Hay, J.F., Winocur, G., Moscovitch, M., 2002. Aging and autobiographical memory: Dissociating episodic from semantic retrieval. Psychol. Aging 17, 677–689. doi:10.1037//0882-7974.17.4.677

Morey, R.D., Rouder, J.N., 2014. BayesFactor: Computation of Bayes factors for common designs [WWW Document]. URL http://cran.r-project.org/package=BayesFactor (accessed 5.5.16).

Nuijten, M.B., Wetzels, R., Matzke, D., Dolan, C. V, Wagenmakers, E.-J., 2015. BayesMed: Default Bayesian Hypothesis Tests for Correlation, Partial Correlation, and Mediation [WWW Document]. URL https://cran.r-project.org/web/packages/BayesMed/index.html (accessed 5.5.16).

Smith, S.M., Jenkinson, M., Johansen-Berg, H., Rueckert, D., Nichols, T.E., Mackay, C.E., Watkins, K.E., Ciccarelli, O., Cader, M.Z., Matthews, P.M., Behrens, T.E.J., 2006. Tract-based spatial statistics: voxelwise analysis of multi-subject diffusion data. Neuroimage 31, 1487–505. doi:10.1016/j.neuroimage.2006.02.024
